# NGAL and Metabolomics: The Single Biomarker to Reveal the Metabolome Alterations in Kidney Injury

**DOI:** 10.1155/2013/612032

**Published:** 2013-03-27

**Authors:** A. Noto, F. Cibecchini, V. Fanos, M. Mussap

**Affiliations:** ^1^Neonatal Intensive Care Unit, Puericulture Institute and Neonatal Section, University of Cagliari, Via Ospedale 119, 09124 Cagliari, Italy; ^2^Department of Laboratory Medicine, University Hospital San Martino, Largo Rosanna Benzi 10, 16132 Genova, Italy

## Abstract

Conditions affecting kidney structure and function can be considered acute or chronic, depending on their duration. Acute kidney injury (AKI) is one of a number of acute kidney diseases and consists of an abrupt decline in kidney function after an injury leading to functional and structural changes. The widespread availability of enabling technologies has accelerated the rate of novel biomarker discovery for kidney injury. The introduction of novel biomarkers in clinical practice will lead to better preventative and therapeutic interventions and to improve outcomes of critically ill patients. A number of biomarkers of functional change and cellular damage are under evaluation for early diagnosis, risk assessment, and prognosis of AKI. Neutrophil gelatinase-associated lipocalin (NGAL) has emerged as the most promising biomarker of kidney injury; this protein can be measured by commercially available methods in whole blood, plasma, serum, and urine. Concomitantly, metabolomics appears to be a snapshot of the chemical fingerprints identifying specific cellular processes. In this paper, we describe the role of NGAL for managing AKI and the potential benefits deriving from the combined clinical use of urine NGAL and metabolomics in kidney disease.

## 1. Introduction

The definition of biomarker makes any characteristic that can be objectively measured and evaluated as an indicator of both normal and pathological processes. This is a general description, which may refer to either a laboratory investigation, carried out in biological samples, or to a functional or imaging test [1]. In medicine, a good biomarker has the ability to increase the performance of the clinical care of a specific disease. Due to this, the significant improvement in the diagnosis, prognosis and/or treatment of the disease has to be demonstrated before accepting a new marker in clinical practice. This is in accordance with the principles of Evidence-Based Laboratory Medicine [2]. Typically, a biomarker can be classified in different subtypes, such as (a) a biomarker that indicates a risk factor, if it identifies a risk of developing a disease; (b) a marker for screening, if it can be used for the assessment of a disease state; (c) a diagnostic index, if it can be used to recognize a state of overt disease; (d) a staging biomarker, if it can be used to stratify patients into different classes of severity of disease; (e) a prognostic index, if it is able to predict the future of the natural history of the morbidity; (f) an index of response to the therapy, if it can be used for monitoring the therapeutic drug. In addition, from the biological point of view, a good “biomarker” should be a protein that originates from the injured cells, and its quantity should be proportional to the mRNA expression. Furthermore, its appearance should be temporally related to the inciting stimulus, and the expression of the biomarker should rapidly decay when the acute phase of injury has terminated [3]. 

## 2. Biomarker of Kidney Damage

Given that acute kidney injury (AKI) is associated with significant morbidity and mortality, and because no specific treatment is available to reverse AKI, early recognition and management is paramount. Taking into account that traditional biomarkers such as serum creatinine are very insensitive and influenced by various extrarenal factors, there is a need to identify new biomarkers of renal cell injury capable to identify additional patients with AKI and the majority of patients at an earlier stage. [4]. In the past, research aimed at evaluating the two main physiological mechanisms underlying the excretory function of the kidney, which are the glomerular filtration and the tubular reabsorption of peptides and proteins. These mechanisms are in fact used to calculate the glomerular filtration rate (GFR); however, they do not always reflect the functional status of the kidney in case of injury [5]. As a matter of fact, a biochemical test should ideally be able to reveal early and specifically the deterioration of the renal function in order to assess the extent of morphofunctional damage. In fact, a correct diagnosis would lead to an appropriate therapy which prevents morbidity and mortality associated with this condition. In the context of renal disease, the use of creatinine, alone is not an accurate measure of renal function since its correlation with the GFR is not linear, its increase over the values of normality occurs after a substantial loss of renal function, and its concentration is reduced in situations such as loss of muscle mass, amputation, chronic organic disease, liver disease, obesity, vegetarian diet, and extremes of age. Moreover, the identification of early AKI is clinically relevant, not by chance that the assessment of risk factors for kidney damage and an early diagnosis of kidney diseases are considered primary targets by the World Health Organization (WHO). Novel urine and serum biomarkers may significantly improve outcome and reduce mortality if they are able to indicate AKI hours after an insult, in comparison with the days it may take serum creatinine to rise substantially. Currently, at least around twenty of candidate biochemical markers of AKI have been studied and proposed in the literature; the most popular investigated biomarker was neutrophil gelatinase-associated lipocalin (NGAL) [5]. 

## 3. Neutrophil Gelatinase-Associated Lipocalin (NGAL)

 Few years ago, the American Society of Nephrology (ASN) in agreement with the National Health Institute (NHI) selected the AKI as area of interest because it was considered an area which needed a more indepth study. An AKI group was created and subsequently divided into several working subgroups with one of these being the biomarkers group. This group was one of the most significant and successful as it discovered a number of genes and proteins related to the early detection of renal damage. NGAL was one of the genes discovered during AKI [6]. NGAL is an ubiquitous protein of 178 amino acids with a molecular mass of approximately 25 kDa and composed of 8 beta sheets that form a shaped structure. It belongs to the family of lipocalins, which are a family of proteins that transport small hydrophobic molecules, such as steroids and lipids [7]. The lipocalins have been associated with many biological processes, such as inflammation, the transport of pheromones, and the synthesis of prostaglandins [8]. NGAL was initially identified by Allen and Venge in 1989 from human neutrophils, and it is now known that it is expressed at low levels in several human tissues, including kidney, trachea, lungs, stomach, and colon [9]. However, its precise role has been only recently clarified by Paragas et al. (2011), who worked on an experimental model of mouse and was able to point out the production of NGAL in case of kidney damage. Initially, the group developed a reporter mouse using a construct formed by the gene of NGAL associated with a luciferase, which enabled the comparison of the NGAL gene and protein expression *in vivo*. They studied the correlation between NGAL mRNA level produced by the kidney and the protein released from the kidney to the urine in real time. By inducing kidney injury experimentally, the timing and the intensity of NGAL mRNA and protein were correlated and dependent on the degree of kidney damage. In addition, they found that the detection of the injury was possible after 3 hours whereas serum creatine rose after about 12 h, and its production was located in the distal convoluted tubule and the collecting duct while the proximal tubule was involved in the process of plasma NGAL reabsorption [3]. In that regard, Portillia et al. (2009) highlighted the role of NGAL as marker of proximal tubule damage in a population of 40 children developing AKI after cardiac surgery. In this study, they were able to correlate the improper function carried by the proximal tubule with the level of NGAL. They also postulated that acute kidney injury resulting from hypoxic condition could cause disruption of megalin-dependent endocytosis in the renal proximal tubule, resulting in the loss of urinary NGAL and suggesting it as a marker of proximal injury [10]. In agreement with the latter, Kuwabara et al. (2009) published a recent work in which, using an experimental animal model of diabetic nephropathy: the STZ-mice (Streptozotocin, 180 mg/kg of body weight), they were able to underline that, after kidney injury, the primary source of NGAL found in the urine was the glomerular filtrate and the cause of this was the proximal tubule damage. In addition, the NGAL mRNA expression in the kidneys of STZ mice was slightly increased compared to control mice. These findings indicate that urinary NGAL is a biomarker that can reflect damage in glomeruli, proximal tubules [11]. However, the role of NGAL does not seem to be yet fully clarified, as showed in a recent paper by Bagshaw et al. (2010), who demonstrated an additional role of NGAL in AKI complicated by sepsis [12, 13]. The experimental design involved the recruitment of 83 patients, half of which had AKI, while the remainder had sepsis-induced AKI. The purpose of that study was to investigate whether there were significant differences between the plasma and urinary NGAL in patients with AKI complicated by sepsis compared with patients with AKI without sepsis. The results of this study showed that although the values of plasma NGAL (pNGAL) and urine NGAL (uNGAL) were on average higher in patients with sepsis, the value of pNGAL was increased within 12 hours in patients with AKI and sepsis compared with patients with AKI and no sepsis. However, results show no significant differences at 24 and 48 hours. As regards to uNGAL, the patients with AKI and sepsis had a higher value within 24 hours in this case too, but there were no significant differences in later times. In conclusion, relative changes in pNGAL and uNGAL may have diagnostic and/or prognostic value and should be evaluated in further investigations. These observations suggest there are differential biomarker patterns in septic AKI that may have clinical relevance and prognostic importance. Another key point about the role of NGAL was provided by a recent paper published in 2010 by Grenier et al. The purpose of their study was to investigate the role of NGAL protein in chronic kidney disease (CKD) [14]. CKD is known to be a major concern for public health around the world since it affects up to 20 million people in the USA and between 2 and 3 million people in Italy. Such studies have shown an unambiguous role of NGAL in the early diagnosis of AKI, but this protein has many other functions, some of which are still not well elucidated. The experimental design organized by the group led by Viau started from a mouse model (FVB/N) that developed renal lesions and with which they were able to demonstrate that the NGAL production not only had a role as kidney injury marker, but itself was involved in some way to the progression of the disease. In fact, the severity of the lesions was significantly reduced in the same experimental mouse model in which the gene NGAL/Lcn2 was silenced (NGAL/Lcn2 −/−). These results point out a new important role of NGAL in the progression of CKD.

NGAL can be measured in whole blood, serum, plasma, and urine by several commercially available analytical immunoassays. They include a whole blood point of care immunoassay (Triage NGAL Test, Biosite-Inverness Medical, Waltham, MA, USA), a fully automated immunoturbidimetric assay optimized for measuring urine NGAL on the Architect analytical platform (Abbott Laboratories, Abbott Park, IL, USA) [15], and an enhanced turbidimetric immunoassay for the measurement of NGAL in urine and EDTA plasma (NGAL Test, BioPorto Diagnostics A/S, Gentofte, Denmark). The latter represents an evolution of a previously exiting manual enzyme-linked immunosorbent assay (ELISA), commercialized by the same manufacturer, and can be adapted for use on a variety of automated clinical chemistry analyzers.

Being NGAL a critical component of innate immunity to bacterial infection, it is also expressed during systemic inflammation and sepsis, increasing in the blood stream and, in turn, in urine. Moreover, during systemic inflammation and sepsis, uNGAL significantly increases because of neutrophils accumulation within the tubular lumen. Consequently, uNGAL can increase (a) as a result of a renal tubular damage; (b) in the course of an acute phase response; (c) as the concomitant presence of sepsis with AKI. On the other hand, three isoforms of human NGAL have been isolated: a 25 kDa monomer, a 45 kDa disulfide-linked homodimer, and a 135 kDa heterodimer consisting of a monomer covalently bound with neutrophil gelatinase, also named matrix metalloproteinase (MMP-9) via an intermolecular disulfide bridge. Neutrophils synthesize the monomer and the homodimer, whereas renal tubular epithelial cells synthesize the monomer and, to some extent, the heterodimer [16, 17]. Theoretically, an “ideal” immunoassay capable to distinguish various molecular forms of uNGAL should permit to assess the origin of uNGAL and, ultimately, the pathological process leading to the changes in uNGAL concentration [17]. As a consequence, we could distinguish sepsis-induced uNGAL from AKI-induced uNGAL excretion. Unfortunately, this “ideal” immunoassay does not exist; the cocktail of antibodies used in the current NGAL immunoassays does not distinguish between the isoforms of the protein, and thus there is a need to develop immunoassays with combination of polyclonal and monoclonal antibodies that preferentially recognize monomeric NGAL originating from kidney tubules, which could, hence, be distinguished from the homodimer and, especially, from the monomeric form synthesized by neutrophils due to the different molecular structure and epitope exposure.

## 4. The New Era of Discovery and Developing Biomarker: The Metabolomics 

The advent of metabolomics, in which all of the metabolites in a given tissue or biological fluid are examined (with the caveat that some metabolites will not be detected in any given experiment), is one of the latest advances in the field of omics. The term metabolome was firstly coined in 1998 by Oliver et al. to designate the set of low molecular weight compounds synthesized by an organism [18]. Afterwards, the first draft of the human metabolome was completed on January 23, 2007, and consisted of a database of about 2,500 metabolites [19]. The word metabolomics means to study a particular set of samples from a holistic rather than reductionist point of view. This omics science uses a series of techniques such as proton nuclear magnetic resonance (^1^H NMR) spectroscopy and gas chromatography-mass spectrometry (GC-MS) which are capable of giving a general profile of all the metabolites present in a set of samples analyzed. The metabolome represents the collection of all metabolites within a biological sample such as urine, blood, and saliva, which are the end products of their gene expression together with the contribution of the environment. From a clinical point of view, the study of the metabolome is comparable to observe a snapshot of a particular biological sample which may reveal new biochemical pattern that could be used for the diagnosis and classification of diseases as well as to improve the understanding of pathophysiological mechanisms. In fact, the metabolomics approach, by providing access to a portion of biomolecular space not covered by other approaches such as proteomics and genomics, offers unique insights into small molecule regulation and signaling. Moreover, this “omics” approach has been successfully used in the fields of physiology, neonatology, pharmacology, toxicology, and nutrition [20–25].

Two strategies configure metabolomics studies: the targeted and the nontargeted approach [26]. The targeted approach is focused on the investigation of several well-defined compounds; it is only used when the target of a drug or disease process is at least partially understood. The nontargeted approach can be defined as a “nonspecific approach”; it allows to investigate both endogenous and exogenous metabolites detectable in a biological fluid or in a tissue. This approach is focused on capturing as much information as possible, providing a functional fingerprint of the physiological and pathological state of the body. Metabolic fingerprinting describes the unbiased analysis of the metabolome by investigating metabolite patterns in different experimental groups with the subsequent classification of these patterns into a fingerprint [27]. Samples can be classified if the metabolite fingerprints differ between groups. Metabolite identification relies on public databases [28]: the Human Metabolome Database (HMDB, the metabolomic equivalent of GenBank). It is an open access database (http://www.hmdb.ca) providing reference to nuclear magnetic resonance (NMR) and mass spectra, metabolite disease associations, metabolic pathway data, and reference to metabolite concentrations for hundreds of human metabolites from several biofluids [29]. In most cases, proton nuclear magnetic resonance (^1^H NMR) spectroscopy and MS-based assays are used for metabolic fingerprinting [30]; these techniques require a well-defined sample preparation. Concisely, ^1^H NMR spectroscopy allows for the simultaneous detection of 20–50 metabolites with an analytical sensitivity ranging 1–10 mmol/L; below this cutoff, the detection and quantification of metabolites is still unreliable, although high field NMR spectroscopy and cryoprobes can improve sensitivity [31]. On the other hand, MS is considered the gold standard in metabolite detection and quantification until now; depending on the metabolite, the sensitivity of MS is in the picomolar and nanomolar range. However, MS should be coupled to an array of separation techniques including gas chromatography (GC) and liquid chromatography (LC); in addition, MS requires longer analytical time (20–60 min for each sample), extensive sample preparation including derivatization, and the limitation to volatile compounds [32]. Other technologies less commonly used for metabolomics are Raman and infrared spectroscopy. Each method has serious drawbacks, such that neither by itself is ideal.

 Metabolomics appears to be extremely attractive for research and clinical purposes, because it offers several advantages over genomics, transcriptomics, and proteomics. Firstly, metabolites vary both quantitatively and qualitatively at any given time. Secondly, metabolic response is extremely rapid, and thus it can be measured within a very short time from the start of the process, while transcriptomics and proteomics are “late signals,” since their response to a challenge may take hours, days, and sometimes weeks. Thirdly, the metabolome communicates with the environment, being an open system, whereas transcriptomics and proteomics strictly detect endogenous changes. Last but not least, despite the very high overall number of endogenous metabolites (~100,000), the number of major metabolites relevant for clinical diagnostics and drug development has been estimated at 1,400–3,000 molecules [33], which means less data to manipulate and interpret, being genes (~25,000), transcripts (~85,000), and proteins (>10,000,000) greatly outnumbered.

## 5. The Metabolomics Approach in Kidney Disease

The recent development of the omics technique also reached the field of kidney disease and seems to be promising in offering new target metabolites that may be considered biomarkers in the next future [34, 35]. In a recent paper, the metabolic difference was investigated between populations of healthy adults born with an extremely low birth weight (ELBW) and those born at term appropriate for gestational age (AGA) [36]. The purpose of that work was to verify the presence of metabolites that might be considered as “biomarkers” that can predict the development of diseases in adulthood. The study was conducted on two groups of individuals, and urine samples were collected aseptically from a group of 18 subjects (8 males and 10 females, mean age 24 years, SD: ± 4.27) born ELBW and a group of other 13 (7 males and 6 females, mean age 25 years, SD: ± 5.15) born AGA. These samples were analyzed using the automated uNGAL method on the Architect platform and then by metabolomics (^1^H-NMR spectrometer Varian 500 MHz, Agilent Technologies). uNGAL concentration showed a significant difference between the ELBW subjects (median 577, range: 32 to 10,224 mg/g urinary creatinine) compared with the AGA group (median 10.6, range: 2.2 to 53, 4 mg/g urinary creatinine) (*P* value < 0.05). In addition, the ^1^H-NMR metabolomics analysis, through a mathematical model, was able to correlate the ELBW metabolic profiles with uNGAL concentration; conversely, uNGAL could not be correlated to AGA. This study, for the first time, demonstrated the relevance of the use of the metabolomics technique together with a novel promising marker of renal damage. It is therefore reasonable to consider that further studies are required about the use of both of these techniques, which may underline the potential prognostic value in case of disease such as the chronic kidney diseases. In fact, this finding may contribute to the identification of people who may develop chronic kidney failure in the near future or in adulthood.

## 6. Conclusion

Increasing knowledge in the science of biology and medicine has accelerated the discovery of novel biomarkers and elucidated their roles in molecular pathways triggered by physiological and/or pathological conditions. Emerging tools, like metabolomics, depend on sophisticated technologies (GC-MS, ^1^H NMR, etc.), which contribute to the sudden development of new biochemical and molecular tests. On one hand, there is a need to develop new predictive approaches for a preventive medicine that allow the prediction of asymptomatic conditions and for tailored therapeutic management. On the other hand, there is an urgent need to translate these developing methods (epigenomics, metabolomics, etc.) and next generation biomarkers (NGAL, KIM-1, etc.) from bench to bedside in order to improve clinical outcome and quality of care. This challenge can be addressed, for example, using a combined approach between the potential of new biomarkers such as uNGAL and the “next generation” metabolomics technique. However, clinical quality specifications (sensitivity, specificity, positive and negative predictive values, etc.) should be addressed as well as the balance between costs and benefits, in term of improved patient outcome per dollar (or euro, etc.) spent.

## Abbreviations

AKI: Acute kidney injury
GFR: Glomerular filtration rate
NGAL: Neutrophil gelatinase-associated lipocalin
NMR: Nuclear magnetic resonance.
